# Imaging-Cytology Correlation of Thyroid Nodules with Initially Benign Cytology

**DOI:** 10.1155/2014/491508

**Published:** 2014-10-13

**Authors:** Shin Hye Hwang, Ji Min Sung, Eun-Kyung Kim, Hee Jung Moon, Jin Young Kwak

**Affiliations:** ^1^Department of Radiology, Research Institute of Radiological Science, Yonsei University College of Medicine, 50 Yonsei-ro, Seodaemun-gu, Seoul 120-752, Republic of Korea; ^2^Department of Biostatistics, Bundang CHA Medical Center, Graduate School of Health and Welfare, CHA University, Seongnam 463-712, Republic of Korea

## Abstract

*Objective*. To determine the role of imaging-cytology correlation in reducing false negative results of fine-needle aspiration (FNA) at thyroid nodules. *Methods*. This retrospective study included 667 nodules 1 cm or larger in 649 patients diagnosed as benign at initial cytologic evaluation and that underwent follow-up ultrasound (US) or FNA following a radiologist's opinion on concordance between imaging and cytologic results. We compared the risk of malignancy of nodules classified into subgroups according to the initial US features and imaging-cytology correlation. *Results*. Among included nodules, 11 nodules were proven to be malignant (1.6%) in follow-up FNA or surgery. The malignancy rate was higher in nodules with suspicious US features (11.4%) than in nodules without suspicious US features (0.5%, *P* < 0.001). When a thyroid nodule had discordant US findings on image review after having benign FNA results, malignancy rate increased to 23.3%, significantly higher than that of nodules with suspicious US features (*P* < 0.001). However, no significant difference was found in the risk of malignancy between the nodules without suspicious US features (0.5%) and imaging-cytology concordant nodules (0.6%, *P* = 0.438). *Conclusions*. Repeat FNA can be effectively limited to patients with cytologically benign thyroid nodules showing discordance in imaging-cytology correlation after initial biopsy, which reduces unnecessary repeat aspirations.

## 1. Introduction 

Fine-needle aspiration (FNA) is the standard method used to determine treatment plans for thyroid nodules. Based on the Bethesda system, the most generally accepted system for reporting thyroid cytology, the “benign” category implies a less than 3% risk of malignancy [1]. Follow-up ultrasound (US) is recommended when the nodule has “benign” cytologic result [2]. Repeat FNA is recommended when a nodule shows significant growth or morphologic transformation with “suspicious” US features on follow-up [3, 4]. However, the practical risk of malignancy in nodules with benign cytology varies in each institute, ranging from 2% to 18% [5], and it has even been reported to have gone up to 62% [6]. Therefore, some investigators recommend routine repeat FNA for thyroid nodules with benign cytology [7, 8].

Considering cost-effectiveness and diagnostic value, repeat FNA has been considered when the nodule shows any suspicious feature on the initial US [9, 10]. However, known US features associated with malignancy show an extremely variable probability of malignancy [11]. Microcalcifications, marked hypoechogenicity, and irregular or spiculated margin show a high risk of malignancy, while solid composition and hypoechogenicity show a relatively low positive predictive value (PPV) [11–13]. Based on these results, each suspicious US feature may not be considered as an equal risk factor for malignancy.

Radiologists specialized in breast imaging have been confronted with the same problem in the core needle biopsy of a breast lesion. Correlation of pathologic results with sonographic findings has been used in some institutions to verify that the lesion was adequately sampled. Discordant benign breast nodules are recommended for rebiopsy to confirm the diagnosis. This approach was suggested owing to the wide range of false negative rates of this category [14]. Based on different malignancy rates of suspicious US features in thyroid nodules and considering approaching steps in management of breast lesions, we conjecture that an imaging-cytology correlation can be a better diagnostic approach for patient management than initial US features in a thyroid nodule with benign cytology. Therefore, we investigated the role of imaging-cytology correlation to reduce the false negative rates of cytology at thyroid nodules as compared with the use of initial US features.

## 2. Materials and Methods

### 2.1. Patients

**Figure 1:**
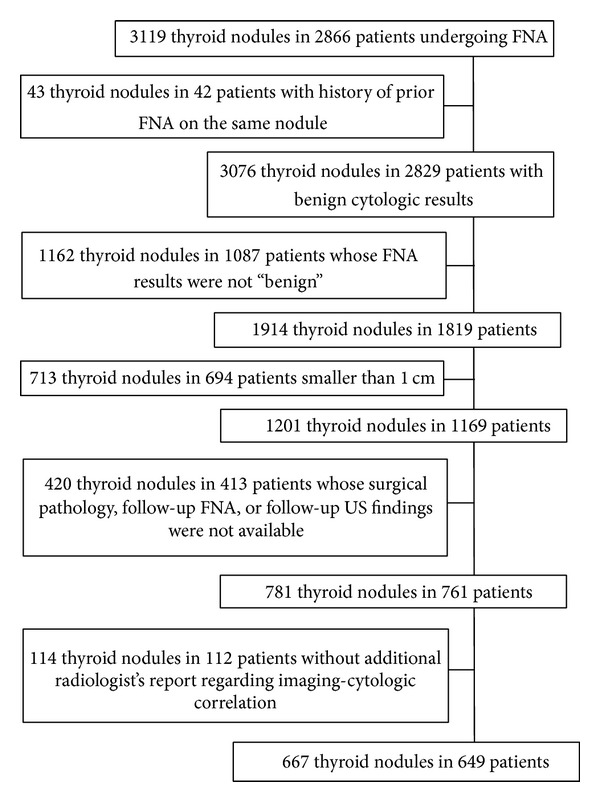
Flow chart of case enrollment.

The Institutional Review Board of the Severance Hospital approved of this retrospective study and required neither patient approval nor informed consent for our review of patients' images and records. However, written informed consents were obtained from all patients for US-guided FNAs (US-FNAs) prior to each procedure as a daily practice. Our institutional registry for the thyroid nodule was settled since 2006 including all patients with thyroid nodules who underwent US examinations and US-FNAs at our institution. From March 2006 to December 2006, 3119 consecutive thyroid nodules in 2866 patients underwent US-FNAs. Among them, we included 667 nodules in 649 patients (men, 83; women, 566), which fulfilled the following criteria: (a) they had no history of prior FNA on the same nodule; (b) they were reported as benign (category II) in the initial FNA. Nodules reported as “nondiagnostic,” “atypia or follicular lesions of undetermined significance,” “follicular neoplasm or suspicious for a follicular neoplasm,” “suspicious for malignancy,” and “malignant” were excluded; (c) they were equal to or larger than 1 cm; (d) they underwent further evaluation such as follow-up US, follow-up FNA, or thyroid surgery. In nodules which had not underwent operation, determinative cytologic reports (category II or category VI) on follow-up US were used as standard reference. If a nodule decreased in size on follow-up US, the nodule was also included as a benign nodule; (e) there were available radiologic reports that included an additional radiologist's opinion about the concordance or discordance between imaging and cytologic results in postbiopsy correlation (Figure 1). Mean age of the patients was 49.1 years (range, 13–87 years). Mean lesion size of the thyroid nodules was 20.7 mm (range, 10–70 mm). Median follow-up of 601 nodules in 584 patients which were included based on followup FNA or US without surgical pathology was 1509 days from the date of initial FNA to the last followup (IQR, 1522 days; range, 172–2744 days). The other nodules were included based on their surgical pathology as a standard reference.

### 2.2. Imaging Methods and Analysis

All US examinations were performed using a 7 to 15 MHz linear array transducer (HDI 5000; Philips Medical Systems, Bothell, Wash) or a 5 to 12 MHz linear probe (iU22, Philips Medical Systems) by 1 of 5 board-certified radiologists with 1 to 12 years of experience in thyroid imaging. All US-FNAs were performed by the same radiologist who performed the US examinations. The nodule size was defined as the largest diameter on US. US features of all thyroid nodules that underwent US-FNAs were prospectively recorded by the previously described methods [19]. US features suspicious for malignancy were determined using previously published criteria from our institution: marked hypoechogenicity, microlobulated or irregular margin, microcalcifications, and taller than wider shape. When overall echogenicity of a nodule was darker than that of the surrounding strap muscle, it was defined as “marked hypoechogenicity” to differentiate it from “hypoechogenicity” based on the parenchymal echogenicity of the thyroid gland. Microlobulated margin meant that a nodule had many small lobular contours on the surface. Microcalcifications were defined as tiny hyperechoic foci either with or without acoustic shadowing. Only calcifications equal to or less than 1 mm in diameter were indicated. If microcalcifications were detected with macrocalcifications, the lesion was considered to have microcalcifications as a worrisome finding. If hyperechoic foci accompanied comet-tail artifacts on conventional US, they were considered as colloids [20]. An anteroposterior to transverse dimension ratio greater than 1 was defined as taller than wider shape.

### 2.3. US-Guided Fine-Needle Aspiration

US-FNAs were performed on either thyroid nodules with suspicious assessment or the largest nodule among nodules with probably benign assessment on US. If there were multiple nodules with suspicious US findings in one patient or if the patient or physician requested a biopsy of a benign-looking nodule coexisting with a nodule showing suspicious US features, FNAs were performed on multiple nodules in one patient. A free-hand biopsy technique was used with either a 23-gauge needle attached to a 20 mL disposable plastic syringe and an aspirator or a 23-gauge needle attached to a 2 mL disposable plastic syringe, depending on the performing radiologist's preference. Each lesion was aspirated at least twice, and the aspirated materials were expelled onto a slide and immediately placed in 95% alcohol for Papanicolaou staining. The remaining materials were rinsed with saline and processed for cell blocking. The cytopathologist was not on site during the biopsy. Five cytopathologists interpreted the slides. Additional special staining was performed according to the requirement of the cytopathologist. An inadequate specimen was defined as less than 6 groups of cells containing more than 10 cells [3]. Adequate specimens were categorized as benign, indeterminate, suspicious for malignancy, or malignant samples.

### 2.4. Imaging-Cytology Correlation and Postaspiration Management

The radiologist who performed FNA routinely reviewed the initial US images within a week of the FNA after the cytologic results were reported. For benign cytologic results, radiologists who performed the US-FNAs decided and reported whether the cytology was concordant or discordant with the imaging findings. As researchers at our institution always try to assess lesions based on their most worrisome finding, the saved images should represent these worrisome US features. Image-cytology correlation was done based on these images. The final conclusion was not derived from the number of suspicious US features but from the subjective decision made by the radiologist who performed the US-FNA. In our institution, “concordant lesions” included some nodules which had suspicious US features on the initial US but were acceptable for the benign cytology in postbiopsy image review as well as the nodules without features suspicious for malignancy on the initial US. Concordant benign thyroid nodules were recommended for follow-up by US after one year. In contrast, “discordant lesions” included nodules which were initially suspected for malignancy on US and were still thought to be suspicious for cancer even after obtaining benign cytology. Repeat FNAs were usually recommended for discordant benign thyroid nodules after 6–12 months [21]. Among the 667 nodules that met all the inclusion criteria, 586 nodules (87.9%, 586 of 667) were reviewed by radiologists who had more than three years of experience in thyroid imaging and FNA whereas the remaining nodules were managed by less experienced radiologist.

### 2.5. Statistical Analysis

We compared the clinical characteristics of patients between benign and malignant nodules by using the *χ* ^2^ test for categorical variables and independent *t*-test for continuous variables. We also compared the risk of malignancy as well as the clinical characteristics between concordant and discordant nodules by using *χ* ^2^ or Fisher's exact test for categorical variables and independent *t*-test for continuous variables. The baseline characteristics were also compared between patients with included and excluded nodules among thyroid nodules equal to or larger than 1 cm with the same methods.

The risk of malignancy was calculated for several subgroups classified according to initial US features and imaging-cytology concordance. Using the generalized estimating equation, we compared the risk of malignancy in thyroid nodules with initially benign cytologic results with those of the remaining subgroups and also compared the risk of malignancy of thyroid nodules among subgroups.

Significance was assumed when the two-sided *P* value was less than .05. Logistic regression analysis was performed to assess the odds ratio for the risk of malignancy. Odds ratios with relative 95% confidence intervals (CIs) were also calculated. Statistical analysis was performed using commercial statistical software (SAS version 9.1, SAS Inc., Cary, NC, USA).

## 3. Results 

Among 667 nodules with initially benign cytologic results, 656 nodules were benign (98.4%) and 11 nodules were malignant (1.6%) based on cytopathology (Table 1). The mean age of patients with malignant nodules was not significantly different from that of patients with benign nodules (*P* = 0.277). Gender of patients was not associated with malignancy (*P* = 0.734). The mean size of malignant nodules (17.6 ± 12.5 mm) was not significantly different from that of benign nodules (20.7 ± 10.1 mm, *P* = 0.315). There were 70 nodules with initial suspicious US features and 597 nodules without initial suspicious US features. The risk of malignancy was higher in nodules with initial suspicious US features (11.4%, 8 of 70) than in nodules without initial suspicious US features (0.5%, 3 of 597; *P* < 0.001, Table 1).

**Figure 2:**
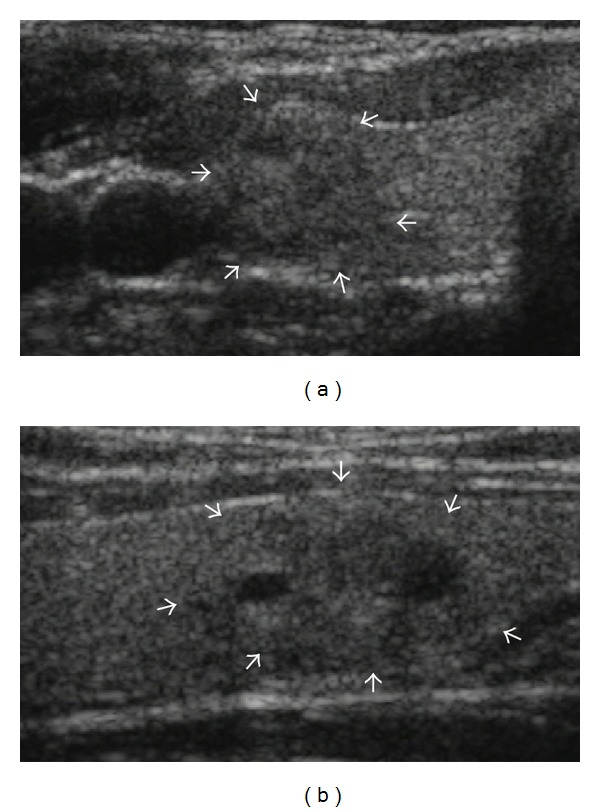
Initially suspicious but concordant nodule after imaging-cytology correlation. US scans ((a) transverse; (b) longitudinal) in a 41-year-old female without remarkable medical history show a 16 mm sized predominantly solid mass (arrows) with microlobulated margin in the lower pole of the right lobe of the thyroid gland. The nodule was taller than wider on transverse scan. The initial cytologic result was adenomatous hyperplasia which was concordant with US findings considering relatively low PPV of these US finding in imaging-cytology correlation after biopsy. A follow-up US was recommended and nodule size gradually decreased from 16 mm to 13 mm with decrease of the cystic portion in follow-up US evaluations until July 2013 without any other significant changes in US features.

**Figure 3:**
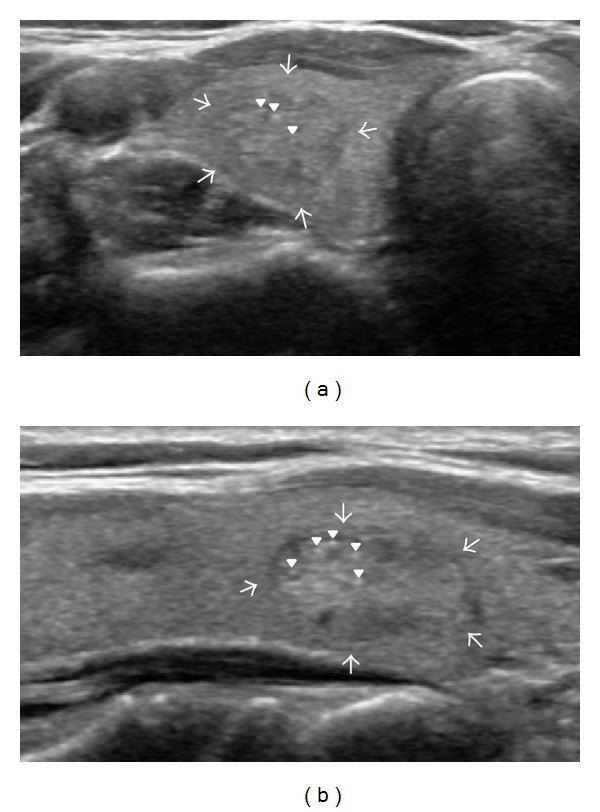
Initially suspicious but concordant nodule after imaging-cytology correlation. US scans ((a) transverse; (b) longitudinal) in a 54-year-old female without remarkable medical history show a 12 mm sized solid mass (arrows) with internal echogenic foci in the lower pole of the right lobe of the thyroid gland. The initial cytologic result was adenomatous hyperplasia which was concordant with US findings in imaging-cytology correlation after biopsy. At the time of imaging-cytology correlation, the echogenic foci (arrowheads) were thought to be related to colloids instead of microcalcifications from psammoma bodies. She underwent surgery (left total and right subtotal thyroidectomy) due to papillary carcinoma in the contralateral lobe of the thyroid gland. The mass in the right lobe was finally confirmed as adenomatous hyperplasia on pathology.

When reviewing US images after initial FNA results were reported, 40 out of 70 nodules which had suspicious features on initial US evaluation were finally concluded as concordant with benign cytology (Figures 2 and 3). Therefore, in 667 nodules with benign cytology, 637 nodules were concordant with cytology, whereas 30 nodules were discordant with benign cytology. The reasons that 40 nodules with revised radiologic diagnosis after imaging-cytologic correlation were initially classified as suspicious nodules were microcalcifications (*n* = 16), microlobulated or irregular margin (*n* = 9), taller than wider shape (*n* = 3), or marked hypoechogenicity (*n* = 1) in order of frequency, respectively, and more than one characteristic of the above features in 11 nodules. Between the concordant and discordant group, gender of the patients was not significantly different (*P* = 0.159). The patients with discordant nodules were significantly older than other patients with concordant nodules (53.5 ± 10.5 years versus 48.9 ± 12.0 years; *P* = 0.038). The mean size of discordant nodules was significantly smaller than that of concordant nodules (16.0 ± 6.6 mm versus 20.9 ± 10.2 mm; *P* < 0.001). The rate of malignancy was significantly higher in the discordant group (23. 3%; 7 of 30) than in the concordant group (0.6%, 4 of 637; *P* < 0.001).

About 44.5% (534 of 1201) of 1 cm or larger nodules with benign cytology in initial FNA were excluded because they had neither standard reference, such as follow-up US, follow-up FNA, or thyroid surgery, nor available radiologist's additional reports regarding imaging-cytologic correlation. The mean age of patients with included nodules was statistically different from the other patients (49.1 ± 12.0 versus 50.7 ± 13.1 years; *P* = 0.033). Patient gender (*P* = 0.392) and mean nodule size (*P* = 0.601) were not significantly different between included and excluded nodules. There were 60 nodules with suspicious findings in the initial US evaluation of excluded nodules (11.2%, 60 of 534), and the proportion was not significantly different from that of included nodules (10.5%, 70 of 667; Table 2).

**Figure 4:**
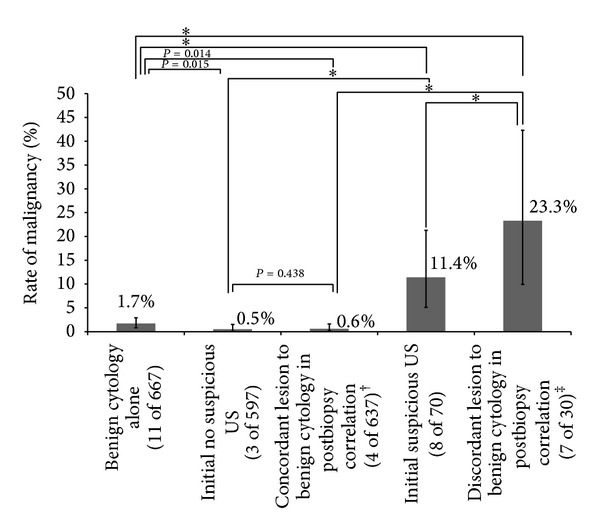
Comparison of malignancy rates in thyroid nodules with benign cytology according to initial US features or imaging-cytology concordance. Error bars for 95% confidence intervals. *P value < 0.001. ^†^Concordant lesions include some nodules which had suspicious US features on initial US but were acceptable for benign cytology in postbiopsy image review as well as nodules without suspicious US features on initial US. ^‡^Discordant lesions include nodules which were initially suspected for malignancy on US and were still regarded as suspicious even after obtaining benign cytology.

When comparing the risk of malignancy between benign cytology alone and each subgroup by a combination of benign cytology with initial US findings or postbiopsy concordance, all combinations had significantly different risk values from cytology alone (Table 3, Figure 4). Also, when comparing the risk of malignancy between discordant lesions and lesions with suspicious features on initial US, the former (23.3%, 7 of 30) was significantly higher than the latter (11.4%, 8 of 70). However, there was no significant difference in the risk of malignancy between concordant lesions (0.6%, 4 of 637) and lesions without suspicious features on initial US (0.5%, 3 of 597; *P* = 0.438) (Figure 4).

## 4. Discussion

Although FNA is a widely used tool for the diagnosis of thyroid nodules, the most significant problem it has is false negative results which bring out misses and delays in treatment of the cancer [22]. Errors in cytologic reports have arisen from the overinterpretation of nondiagnostic specimens as diagnostic ones [23, 24]. Therefore, many reports discussed the differentiation of a nondiagnostic specimen from a diagnostic one in the cytologic interpretation of thyroid FNA [1, 3]. Diagnostic errors of thyroid FNA can also be caused by the mistakes of cytopathologists and the inherent nature of thyroid nodules due to overlapping cytologic criteria among hyperplastic adenomatoid nodule in goiter, follicular adenoma, well-differentiated follicular carcinoma, and follicular variant of papillary carcinoma [25]. Moreover, reported false negative rates are variable among institutions and operators due to variable sampling skills [5, 6].

Several guidelines recommend follow-up US in thyroid nodules with benign cytology unless the nodule shows significant growth or morphologic change in follow-up US [1, 3, 4]. However, it has been argued that follow-up might be not enough in some nodules because of the inevitable false negative diagnosis and the possible risk of delayed treatment [6, 22]. To reduce false negative results of thyroid FNAs, there have been two suggested approaches; first, routine repeat FNA in thyroid nodules with benign cytology [26, 27] and, second, selective repeat FNA [8, 10, 28, 29]. In the aspect of cost-effectiveness, it is more rational to consider performing follow-up FNA selectively for nodules with a high-risk of malignancy rather than performing a total inspection of cytologically benign nodules in initial FNA. Based on several reports, the rate of malignancy in benign thyroid nodules with suspicious US features was 3.7–47.1% which was significantly higher than that of benign thyroid nodules without suspicious US features (Table 4) [9, 15–18]. Although the US criteria applied to each study had subtle differences, initial US features may be reliable factors in determining whether to repeat FNA or not [17].

Going one step further from simply matching cytologic results against imaging findings evaluated before biopsy, the postbiopsy correlation of US features with cytologic results could be an alternative in determining whether the nodule should be reaspirated to confirm its cytology or not. Imaging-pathologic correlation after biopsy has been found to be useful in validating biopsy results of breast lesions, and discordance has been suggested as an indication for excision because of its higher upgrade rate than that of concordant lesions [30–32]. However, there has been no organized study that applies imaging-cytology correlation to patient management and considers how to accept results of postbiopsy correlation in regard to reducing false negative diagnosis in thyroid nodules.

In this study, 1.6% of nodules with benign cytology in initial FNA were finally proven to be malignant. As expected, the malignancy rate of thyroid nodules (11.4%) with suspicious features on initial US was significantly higher than that of nodules (0.5%) without suspicious features on initial US, and the malignancy rate of nodules (23.3%) with discordant imaging findings was also significantly higher compared to concordant nodules (0.6%) in postbiopsy imaging-cytologic correlations. Furthermore, the rate of malignancy was higher in the nodules showing imaging-cytology discordance compared to nodules showing suspicious feature on the initial US. However, there was no significant difference in the risk of malignancy between concordant nodules in postbiopsy correlation and nodules without suspicious features on initial US. This result lets us conclude that imaging-cytology correlation is a more effective approach than using initial US features alone when deciding follow-up management in patients with cytologically benign thyroid nodules without a statistical increase in missing malignancy.

In this study, 40 of 70 nodules with suspicious features on initial US were determined as concordant with benign cytology after postbiopsy imaging-cytology correlation. This change can be explained by the subjective nature of US evaluation. Although many descriptions of each suspicious US feature are present, interobserver and intraobserver variability still exist for the US assessment of thyroid nodules. Among US characteristics, margin and calcification showed relatively less consistency between observers [33] and nodules in most patients whose radiologic assessments were changed after obtaining benign cytology were initially assumed as suspicious nodule due to calcification (16 of 40), margin (9 of 40), or multiple features (11 of 40) including them in our study. Also, there have been difficulties in deciding whether a thyroid nodule shows echogenic spots on US. Echogenic spots can be due to microcalcifications related to cancer or crystals related to colloids [34]. Therefore, postbiopsy imaging-cytology correlation can be a good diagnostic approach in deciding whether to repeat FNA or not at a thyroid nodule with benign cytology.

There were several limitations to this study. First, some nodules were excluded in analysis despite having benign cytologic results due to loss of follow-up and absence of additional reports. Selection bias may be unavoidable. However, the initial US assessment was not significantly different between included nodules and excluded nodules which were 1 cm or larger with benign cytology in the initial FNA. Second, interobserver and intraobserver variability among radiologists are possible in the interpretation of US images and among cytologists, especially when reviewing follicular lesions. Third, there might be a bias arising from the postbiopsy review process itself which was based on saved images instead of on a review in real-time US. Although we always tried to save any images showing worrisome US findings and the postbiopsy review was preferably done within a week of biopsy by the performer, an observer bias might not have been completely removed from the final results. Fourth, suspicious US features such as calcification, margin, vascularity, and echogenicity have been differently applied to thyroid nodules by various guidelines and different institutions. Therefore, the result of this study needs to be validated in other institutions. Fifth, most (87.9%) of the nodules in this study were reviewed by highly experienced radiologists in thyroid imaging. Therefore, the results may not be reproducible in other institutions.

## 5. Conclusions

Repeat FNA can be effectively limited to patients with cytologically benign thyroid nodules showing discordance in imaging-cytology correlation, which reduces unnecessary repeat aspirations as well as decreasing false negative results.

## Figures and Tables

**Table 1:** Baseline characteristics of 667 thyroid nodules with benign cytology.

Reference standard	Benign	Malignant	*P* value
Number of nodules	656	11	
Mean age (years)∗	49.1 ± 12.0	53.0 ± 11.4	0.277
Gender			0.734
Male	82 (12.5)	1 (9.1)	
Female	574 (87.5)	10 (90.9)	
Mean nodule size (mm)∗	20.7 ± 10.1	17.6 ± 12.5	0.315
US final assessment before FNA			<0.001
Probably benign	594 (90.5)	3 (27.3)	
Suspicious malignant	62 (9.5)	8 (72.7)	

FNA: fine-needle aspiration.

Data in parentheses are percentages.

∗Data are the means ± standard deviations.

**Table 2:** Comparison of baseline characteristics of nodules according to inclusion criteria among 1 cm or larger 1201 thyroid nodules with benign cytology.

	Included nodules	Excluded nodules	*P* value
Number of nodules	667	534	
Mean age (years)∗	49.1 ± 12.0	50.7 ± 13.1	0.033
Gender			0.392
Male	83 (12.4)	76 (14.2)	
Female	584 (87.6)	458 (85.8)	
Mean nodule size (mm)∗	20.7 ± 10.1	21.0 ± 10.6	0.601
US final assessment			0.709
Probably benign	597 (89.5)	474 (88.8)	
Suspicious malignant	70 (10.5)	60 (11.2)	

Data in parentheses are percentages.

∗Data are the means ± standard deviations.

**Table 3:** Risk of malignancy according to initial US features and imaging-cytologic correlation in thyroid nodules with benign cytologic results.

	Number of nodules	Number of malignant nodules	Risk of malignancy (%)
Benign cytology alone	667	11	1.6 (0.8, 2.9)
Initial no suspicious US	597	3	0.5 (0.1, 1.5)
Initial suspicious US	70	8	11.4 (5.1, 21.3)
Concordant lesion to benign cytology in postbiopsy correlation	637	4	0.6 (0.2, 1.6)
Discordant lesion to benign cytology in postbiopsy correlation	30	7	23.3 (9.9, 42.3)

Data in parentheses are 95% confidence intervals.

**Table 4:** Reported rate of malignancy in nodules with benign cytology according to US finding.

	Total number of nodules	Rate of malignancy (%)			Suspicious US features
		Overall	Suspicious US	No suspicious US	
Kwak et al. [9]	1343	1.9 (26/1343)	20.4 (19/93)	0.6 (7/1250)	Marked hypoechogenicity, microlobulated or irregular margin, microcalcification, and taller than wider shape
Koike et al. [15]	168	11.9 (20/168)	47.1 (8/17)	7.9 (12/151)	Ill-defined margin, irregular shape, solid echo structure, heterogeneous internal echogenicity, hypoechogenicity, presence of calcification, absence of halo, and invasion of adjacent organs
Lee et al. [16]	560	1.1 (6/560)	3.7 (4/108)	0.4 (2/452)	Marked hypoechogenicity, microlobulated or irregular margin, microcalcification, and taller than wider shape
Maia et al. [17]	35	28.6 (10/35)	38.5 (5/13)	22.7 (5/22)	Hypoechogenicity, microcalcification, border irregularity, and central flow by Doppler study
Choi et al. [18]∗	700	1.7 (12/700)	4.7 (8/169)	0.8 (4/531)	Marked hypoechogenicity, not well-defined margin, microcalcifications, and taller than wide shape

Data in parentheses are numbers used to calculate percentages.

∗Multicenter study from 7 university-affiliated hospitals.
